# Optimizing the microwave-assisted hydrothermal extraction of pectin from tangerine by-product and its physicochemical, structural, and functional properties

**DOI:** 10.1016/j.fochx.2024.101615

**Published:** 2024-07-03

**Authors:** Imed E. Benmebarek, Diego J. Gonzalez-Serrano, Fatemeh Aghababaei, Dimitrios Ziogkas, Rosario Garcia-Cruz, Abbas Boukhari, Andres Moreno, Milad Hadidi

**Affiliations:** a Laboratory of Organic Synthesis, Modeling and Optimization of Chemical Processes, Department of Chemistry, Faculty of Sciences, Badji Mokhtar-Annaba University, BP 12, 23000 Annaba, Algeria; b Department of Inorganic, Organic and Biochemistry, Faculty of Chemical Sciences and Technologies, University of Castilla-La Mancha, 13071 Ciudad Real, Spain; c Ahora Health, Scientific Park of Madrid, 28049 Madrid, Spain; d Institute of Physiological Chemistry, Faculty of Chemistry, University of Vienna, Vienna 1090, Austria

**Keywords:** Tangerine peel waste, Pectin extraction, Optimization, Techno-functional properties, Antioxidant activity

## Abstract

Microwave-assisted hydrothermal extraction (MAHE) was optimized using a Box-Behnken design (BBD) of the response surface methodology (RSM) for optimal recovery of pectin from tangerine peel (TPP). The effects of three factors (pH, irradiation time and temperature) on extraction yield (EY), galacturonic acid content (GAC) and degree of esterification (DE) of pectin were investigated. The optimal extraction conditions were as follows: pH 1.7, irradiation time 12 min and temperature 109 °C. Under these conditions, the EY, GAC and DE were 30.4, 72.3 and 45.2%, respectively. The low methoxyl content of MHAE (45.2%) compared to CE is confirmed by the ^1^H NMR and FTIR spectra, and the emulsifying activity is 57.65% and 50.56% for CE and MHAE, respectively. The total phenolic content (TPC) of pectin produced using MAHE is 41.2 mg GAE/g, thus indicating higher antioxidant properties compared to pectin produced with CE, which had a TPC of 38.4 mg GAE/g. In addition, the X-ray diffraction (XRD) and surface morphological analysis (SEM) results showed that TPP had a rough surface and crystalline structure. Overall, our findings show that TTP from MAHE can be used as a natural antioxidant ingredient in the functional food and pharmaceutical industries.

## Highlights

• Microwave-assisted hydrothermal extraction is a novel technique for pectin extraction.
• Physicochemical, structural, and functional properties of pectin were studied.
• Extracted pectin had a rough surface and crystalline structure.
• Isolated pectin can be used as a natural antioxidant ingredient.

## 1 Introduction

The common tangerine (*Citrus reticulata*) is an easy-to-peel citrus fruit well-known for its delicate taste, nutritional value, bioactive compounds content, and uses in beverages, desserts, and pharmaceutical products. With a global production of >30 million tons in 2020, new processes are needed to utilize the by-products of this sector (Shorbagi et al., 2022), especially tangerine peel (TP), which comprises 40–50% of the fruit's weight and has a pectin content of almost 13%, thus making it a valuable by-product for extraction of the latter (El Barnossi et al., 2021). Pectin is a valuable anionic biopolymer, the structure of which includes a variety of complex polysaccharides, mainly rhamnogalacturonan I and homogalacturonan, with poly (1,4)-D-galacturonic acid residues as side chains (Petkowicz et al., 2017). The degree of esterification (DE) is one of the most important factors in the application of pectin, and there are two distinct types of low- and high-methoxyl pectin with degrees of esterification less than or >50%, respectively (Kazemi et al., 2019).

Pectin is rapidly becoming a versatile hydrocolloid with a wide range of uses, such as in therapeutic treatments (encapsulation of prebiotics and probiotics, drug delivery, and wound and cancer therapy), but also for industrial food uses like 3D printed foods, natural food additives (E440), water-holding, control of dietary fat and energy uptake, and bio-nano-packaging (Adetunji et al., 2017). The worldwide consumption of pectin was 70,000 tons in 2020, with a value of $18/kg, and novel green methods will soon be applied to produce pectin industrially (Ciriminna et al., 2022). The search for new pectin sources seems promising and, in this regard, industrial waste by-products have emerged as an intriguing option for the extraction of pectin (Adetunji et al., 2017). Thus, although most plants contain pectin, the majority of pectic polysaccharides used in the food industry come from citrus fruits and apple by-products due to their desired capacity for gel formation. However, alternative sources, such as passion fruit, pumpkin, mango, and soy hull, are currently being studied (Donaghy & Mckay, 1994; Liew et al., 2014).

Most commercial pectin is extracted using acidic aqueous solutions at elevated temperatures, generally 85 °C, employing conventional heating together with mineral acids such as HCl or HNO_3_, even though these reagents generate ecological concerns (Picot-Allain et al., 2022). Despite its widespread use, conventional heating has the major disadvantage of long extraction times and high solvent consumption (Chen et al., 2021; Hadidi, Orellana Palacios, et al., 2023). Time, temperature, pH, solid–liquid ratio, granulometry, and the form of the raw material also affect the extraction, not necessarily just the yield but also the monosaccharide composition and the physicochemical properties (Mao et al., 2019). Microwave-assisted extraction has demonstrated potential in terms of saving time, energy, and solvent consumption (Dranca et al., 2021; Picot-Allain et al., 2022). More specifically, microwave-assisted hydrothermal extraction is an environmentally friendly method that allows the water to be heated to high temperatures quickly in less time. Indeed, this method has been used to extract pectin from mango peel waste (Matharu et al., 2016) and the green tide algae *Ulva prolifera* (Yuan et al., 2018).

In this research, hydrothermal microwave-assisted extraction (MAHE) and conventional extraction (CE) were used to extract pectin from TP. Thus, MAHE was developed and optimized using Boxh–Benhken Design (BBD). This allowed the recovery of pectin from TP in a safe way, with microwave irradiation as an energy source, under hydrothermal conditions (fixing the pressure and temperature), and citric acid as catalyst, which is environmentally friendly. To the best of our knowledge, this is among the best extractions of pectin using an environmentally friendly process. The pectin extracted on a laboratory scale was characterized by FT-IR and ^1^H NMR spectroscopy and TGA. The physicochemical, structural, and functional properties of the pectin obtained under optimal extraction conditions were determined.

## 2 Materials and methods

### 2.1 Materials

Fresh tangerines were purchased from a local supermarket in Ciudad Real (Spain). The peels were cleaned, cut into small pieces, and dried at 70 °C. The dried peels were then placed in a blender and sieved through a 40-mesh screen to eliminate any non-powdered particles. The resulting powder was kept in a dry, dark place in preparation for the subsequent experiments. Citric acid (99%), sulfuric acid (95–97%), hydrochloric acid (37%), trifluoroacetic acid (TFA, 99%), Folin–Ciocalteu phenol, sodium hydroxide (NaOH, 99%), ethanol (96%), ascorbic acid (AA), carbazole, sodium azide (NaN_3_), sodium chloride (NaCl), gallic acid, sodium carbonate, D-(+)-galacturonic acid, D_2_O, and 2,2-diphenyl-1-picrylhydrazyl (DPPH) were acquired from Merck Chemical Co. (Darmstadt, Germany).

### 2.2 Pectin extraction

#### 2.2.1 Conventional extraction (CE)

Pectin was extracted by hot acidic extraction using a water bath based on the method described by Chaharbaghi et al. (2017), with minor modifications. First, 5 g of dry powdered TP was diluted with distilled water (powder to solvent ratio 1:20 *w*/*v*), and 1 M citric acid with 6 M HCl was used to adjust the pH to the required value (pH 1). The mixture was then placed in the water bath at 90 °C for 2 h. After extraction at room temperature, the mixture was separated from the reaction products.

#### 2.2.2 Microwave-assisted hydrothermal extraction (MAHE)

The MAHE process was conducted using a Milestone SynthWave microwave system. This system consists of a 1-L batch reactor made of high-grade stainless steel that is water-cooled and is able to withstand high temperatures and pressures. The reactor is connected to a magnetron, which generates microwave radiation, via a waveguide. The pressure vessel, constructed from special stainless steel, is securely locked with a clamping device. Microwave power is regulated using a proportional integral derivative (PID) controller. The internal temperature and pressure are monitored and controlled using a thermowell and a pressure sensor, respectively. For the MAHE experiments, the power was adjusted to achieve the desired reaction temperature rapidly and accurately. A power of 1500 W was utilized, resulting in the generation of approximately 1500 W of microwave energy at a frequency of 2450 MHz (Lorente et al., 2019).

In the experiments, the reactor was filled with 3 g of TP (test material) and 60 mL of different solutions with pH values of 1, 1.5, and 2. The experiments were conducted at a pressure of 5 bar using nitrogen gas (N_2_) as the medium. The aim was to investigate the effects of reaction temperature and time on the process. The temperature ranged from 70 to 110 °C, and the reaction time varied from 4 to 12 min. After completion of the reaction, the temperature was gradually reduced. The microwave cavity, which is water-cooled, was connected to a chiller containing water at approximately 5 °C to rapidly lower the temperature. Subsequently, the pressure was released to obtain the reaction products.

#### 2.2.3 Pectin purification

CE and MAHE were used to extract the TPP, and the procedure for both methods was similar: an aqueous solution mixed with peel residue. The mixture was filtered with a muslin cloth and centrifuged at 8000*g* for 20 min. A comparable amount of 96% ethanol was used to precipitate the supernatant, and it was kept at 4 °C overnight. The precipitated pectin was then separated by centrifugation (8000 *g*, 20 min), and the pectin pellet obtained was purified by washing with ethanol (2 × 100 mL). The estimated extraction yield (EY) was calculated using **Eq.** (1) (on a dry basis):

(1) $$\mathrm{PY}\%=\frac{\mathrm{weight of}\;\mathrm{TPP}\;\left(g\right)}{\mathrm{weight of}\;\mathrm{TP}\;\left(g\right)}\;x\;100$$

### 2.3 Experimental design

To obtain the optimal ratio, one factor was varied at a time in this method. The liquid-solid ratio was one of the variables varied (5, 10, 20, 30, 40, and 50 *v*/*w*) while the others remained the same (pH 1.5, irradiation time = 8 min, and temperature = 90 °C). After selecting the best ratio, this variable was maintained in all subsequent experiments. However, the impact of the various variables (pH, irradiation time, and temperature) on the extraction yield (EY), galacturonic acid (GA) content, and degree of esterification (DE) of TPP was assessed using a Box–Behnken design (BBD) with three variables at three levels (Table 1). Design Expert 11.0 and Excel were used for all statistical calculations and visualizations.

**Table 1:** Experimental Box–Behnken design for the yield, GA, and DE of TPP.

Run		Independent factors			Experimental results
	pH (X_1_)	Time (X_2_)	Temperature (X_3_)	Yield (%)	GA (%)
1	1.5	110	4	24	55
2	1.5	90	8	27.3	61.1
3	2	110	8	25	74.3
4	2	90	12	26.2	76.6
5	1.5	70	4	27	48
6	1	90	12	26	55.5
7	1.5	110	12	32	65.7
8	1	110	8	25.3	45.4
9	1	90	4	23	40
10	1.5	90	8	28	62.2
11	1.5	90	8	27	63.7
12	2	90	4	25	68
13	1	70	8	20	35.7
14	2	70	8	23	54
15	1.5	70	12	24	53

### 2.4 Galacturonic acid content (GAC)

The GAC was measured based on the method of Rodsamran and Sothornvit (2019) with minor modifications. Firstly, 6 mL of concentrated sulfuric acid was combined with 1 mL of pectin solution (10 mg/100 mL deionized water) and left for 20 min. Then, 0.6 mL of a 0.15% carbazole solution (in pure ethanol) was added, and the mixture was allowed to stand for 2 h until a pink color appeased. A spectrophotometer (Shimadzu UV–Visible 1800, Tokyo, Japan) was used to measure the absorbance at 520 nm. The GAC was determined using a D-(+)-galacturonic acid standard curve (0–100 mg/mL) and was represented as milligrams per gram of pectin (mg/g).

### 2.5 Degree of esterification (DE)

A ^1^H NMR spectroscopy technique, which was adopted from Müller-Maatsch et al. (Müller-Maatsch et al., 2014), was used to determine the DE of pectin. Internal standard (TSP, 0.2 mg/mL in D_2_O) and TP pectin (30.0 mg) were incubated with NaOH (0.4 M, 1.0 mL) in D_2_O for 2 h. The liquid was then placed into NMR tubes after being centrifuged (5810R, Eppeendorf, Germany) and filtered. ^1^H NMR spectra were recorded at 25 °C and a frequency of 500 MHz using a Bruker AV 500 spectrometer (Karlsruhe, Germany). By manually integrating the related signals, acetic acid, methanol, and ferulic acid were determined quantitatively (1.980 ppm for acetic acid, 3.378 ppm for methanol, and 6.233 ppm for ferulic acid) and compared with the TSP area (0 ppm). Integrals were transformed into mass values (W_x_, mg) using Eq. 2, as reported previously.

(2) $$\mathrm{Ax}\times \frac{{\mathrm{EW}}_{X}}{W_{X}}=A_{\mathrm{TSP}}\times \frac{{\mathrm{EW}}_{\mathrm{TSP}}}{W_{\mathrm{TSP}}}$$

A_x_ is the analyte spectral region, A_TSP_ is the spectral region of the internal standard, EW_x_ is the analyte equivalent weight, EW_TSP_ is the internal standard equivalent weight, EW = (molecular weight/signal hydrogen number), W_TSP_ is the weight of the reference standard TSP in milligrams, and W_x_ is the weight of the sample, based on the amount of GA in the sample, as measured using the UV technique, in milligrams. Eq. (3) was used to determine the DE:

(3) $$\mathrm{DE}\%=\frac{\mathrm{mol}\;\mathrm{of methanol}+\mathrm{mol}\;\mathrm{acetic acid}+\mathrm{mol}\;\mathrm{ferulic acid}}{\mathrm{mol}\;\mathrm{of galcturonic acid}\;}\times 100\%$$

### 2.6 Protein content (PC)

The Kjeldahl method was used to determine the protein content. In a temperature-resistant tube at 400 °C, 1 g of extracted pectin was digested in 15 mL of concentrated sulfuric acid with a Kjeldahl catalyst (6.25% in CuSO_4_5H_2_O, Merck, Darmstadt, Germany). After digestion, the cooled digest was diluted with distilled water. The solution was treated with 60 mL NaOH (35%) and then steam distilled to separate the volatile NH3 from the remaining components. The condensed NH3 was then collected in 1% dilute boric acid. The nitrogen was measured after titration with HCL (0.1 M) and the protein concentration was determined using a conversion factor of 6.25 (Hadidi, Hossienpour, et al., 2023; Orellana-Palacios et al., 2022).

### 2.7 Total phenolic content (TPC)

The method of Chaouch et al. (2015) with some modifications, was used to determine the TPC for TPP extracted under optimal conditions and by conventional pectin extraction. After the preparation of a 0.5 mL pectin sample (10 mg/mL), 2.5 mL Folin-Ciocalteu reagent (10% *v*/v) and 2 mL sodium carbonate solution (7.5 mg/mL) were added in that order. The absorbance of the mixture was measured at a wavelength of 750 nm after 2 h at room temperature. Gallic acid serves as a reference, and the TPC of the sample was calculated in terms of mg of gallic acid equivalents per gram of tangerine pectin (mg GAE/g).

### 2.8 DPPH of radical scavenging activity

The antioxidant activity of the TPP extracted was studied using the procedures described in Chaouch et al. (2015) with a few modifications. Thus, various concentrations of a 1 mL sample (1–50 mg/mL) and 4 mL of a dilute DPPH solution in methanol (100 μM) were added, and the mixture was thoroughly mixed and kept at room temperature for 45 min before measuring the absorbance at 517 nm with a Spectrum SP-UV500DB UV–Vis spectrophotometer. Ascorbic acid (AA) at comparable concentrations was utilized as a standard. A lower absorbance for the samples indicates high antioxidant activity. The following equation was used to obtain the DPPH inhibitory percentage.

(5) $$\mathrm{DPPH}\;\left(\%\right)=\left[1-\left(\frac{A\;\mathrm{sample}}{A\;\mathrm{control}}\right)\right]\times 100$$

### 2.9 Nuclear magnetic resonance (NMR) spectra

For this experiment, the TPP obtained under ideal conditions (10 mg) was dehydrated and dissolved in D_2_O (500 μL), then the ^1^H NMR spectrum was recorded at a temperature of 23 °C using a Bruker AV500 spectrometer (Karlsruhe, Germany). A total of 32 scans were gathered, with a relaxation delay of 1 s and an acquisition time of 4.09 s.

### 2.10 Fourier transform-infrared (FT-IR) analysis

FT-IR spectra were recorded in the 4000–600 cm^−1^ range, with a frequency of 4 cm^−1^, for the TPP obtained under optimal conditions, after dehydration, using a Bruker FT-IR Tensor 27 spectrometer (Billerica, Massachusetts, USA) (Majidiyan et al., 2022).

### 2.11 Thermogravimetric analysis (TGA)

TGA (TGA 550, TA Instruments, New Castle, DE, USA) was used to test the heat resistance of the pectin extracted using the CE and MAHE techniques. This technique is similar to that utilized by Hadidi et al. (2020). Thus, 7 mg of pectin was weighed into a platinum pan and heated at a rate of 10 °C/min up to 900 °C under an argon flow (50 mL/min).

### 2.12 Equivalent weight (Eq.W) and methoxyl content (MC)

The method of Israel et al. (2019) was used to measure the equivalent weight. Thus, 0.5 g of TPP was mixed in 100 mL of distilled water qt 25 °C and stirred well for 2 h. Six drops of phenol red were used as an indicator, then 1 g of sodium chloride (NaCl) was added and the mixture titrated with 0.1 mol/L of NaOH. Finally, the following equation was used to calculate Eq.W:

(6) $$\left(\mathrm{Eq}.W\right)=\frac{1.000\times \mathrm{pectin powder}\left(g\right)\;}{\mathrm{NaOH concentration}\;\left(N\right)\times \mathrm{NaOH volume}\;\left(\mathrm{ml}\right)}$$

The neutralized solution obtained when calculating Eq.W was also used to calculate the MC. Thus, 25 mL of 0.25 M NaOH was added to the neutralized solution, which was then allowed to stand at room temperature for 30 min before being mixed with 25 mL of 0.25 M HCl and titrated with 0.1 M NaOH. The following equation was used to determine the MC.

(7) $$\mathrm{MC}\;\left(\%\right)=\frac{\mathrm{NaOH concentration}\;\left(N\right)\times \mathrm{NaOH volume}\;\left(\mathrm{ml}\right)\times 3.1}{\mathrm{pectin powder}\;}$$

### 2.13 Swelling capacity (SW)

The pectin samples were placed in pH buffer solutions (1.4, 5.4, 7.4, and 9.4) at room temperature for 24 h. They were then wiped with absorbent paper to remove any remaining water that might have been present. The swollen samples were vacuum-dried at 35 °C to constant weight. The following equation was used to determine the percentage swelling as per the technique explained in M. Kumar et al. (2010):

(8) $$\mathrm{SC}\%=\frac{w_{s}-w_{D}}{w_{D}}\times 100$$

W_s_ is the weight of the substance after swelling, and W_D_ is the weight of the drying test sample.

### 2.14 Emulsifying properties

The emulsion activity (EA) and emulsion stability (ES) of TPP were determined for each extracted sample (obtained directly under optimal and conventional conditions) using the technique described by Hosseini et al. (2016). Thus, a combination of 5 mL sunflower oil, 5 mL TPP solution (0.5% *w*/*v*), and 0.02% sodium azide was sonicated for 5 min before centrifugation (4000 *g* for 5 min). Finally, EA was estimated as follows:

(9) $$\mathrm{EA}\left(\%\right)=\frac{V_{E}}{V_{T}}\times 100$$

where V_E_ and V_T_ are the volume of the emulsion layer and the total volume, respectively.

ES maintained for 30 min at 80 °C and centrifuged at 5000 *g* for 5 min was assessed as follows:

(10) $$\mathrm{ES}\left(\%\right)=\frac{V_{R}}{V_{I}}\times 100$$

where V_R_ denotes the remaining emulsion volume and V_I_ the original emulsion volume (Bayar et al., 2017).

### 2.15 X-ray diffraction (XRD)

The XRD patterns of TPP samples extracted under optimized MAHE and CE conditions were measured using an X-ray diffractometer (Philips, Amsterdam, Netherlands). The pectin sample was scanned with a diffraction angle (2*θ*) ranging from 10° to 80°. The step size was 0.05° and the step time was 1 s (Hesami et al., 2022).

### 2.16 Morphology

The morphological analysis of pectin was performed using the approach of Wongkaew et al. (2020), with slight changes. Thus, a scanning electron microscope (SEM) was used to examine the powder morphology of pectin powder. Before the analysis, pectin was sieved using 60 mesh sieves. The powder was then applied to circular metal stubs, which were wrapped in double-sided sticky carbon tape, then gold sputter-coated. SEM images were captured at a magnification of 10.00 KX at an accelerating voltage of 2.00 kV.

## 3 Results and discussion

### 3.1 Statistical analysis and optimization process

In the microwave-assisted hydrothermal extraction (MAHE) of tangerine peel pectin (TPP), an optimal ratio of 1:20 (*v*/*w*) was initially selected. This ratio was then maintained while conducting 15 runs using the Box–Behnken design (BBD) to optimize the remaining three parameters: pH, irradiation time, and temperature (as shown in Table 1). The lowest and highest extraction yields (EY) 20% and 32% were obtained in runs 13 and 7, respectively). Additionally, the galacturonic acid content (GAC) ranged from 35.7% (run 13) to 76.6% (run 4), while the degree of esterification (DE) varied from 32.4% (run 6) to 74% (run 14). Using the anticipated statistical model (Eqs. (8), (9), (10)), the optimal extraction conditions were measured to be pH 1.7, an irradiation time of 12 min, and a temperature of 109 °C. Based on this model, the predicted values for extraction yield (EY), GAC, and DE were 31.3%, 73.3%, and 42.67%, respectively. To validate the model, it was tested experimentally three times under the specified conditions, with the results confirming its high accuracy. The optimal TPP extraction yields, GA, and DE were found to be 30.4 ± 0.6%, 72.3 ± 2.48%, and 45.2 ± 2.74%, respectively.

The results obtained highlight the remarkable efficiency of the MAHE technology in terms of its short processing time, low energy consumption, and high extraction yield (EY) and galacturonic acid content (GA). Notably, the TPP extraction yield achieved using MAHE was found to be higher than the values reported in previous studies for pectin extracted from dragon fruit, apple pomace, pistachio green hull, and orange peel using a simple microwave-assisted extraction method. Specifically, the pectin yields for those materials were reported to be 7.50%, 15.75%, 18.13%, and 28.07%, respectively, this demonstrates the enhanced performance of MAHE for extracting pectin from tangerine peel (Kazemi et al., 2019; K. Kumar et al., 2021; Liew et al., 2014). The MAHE yield of TPP also exceeded the results obtained using optimized normal microwave-assisted extraction (MAE; 28.8%) with other citric fruits (Hosseini et al., 2016). Table 2 lists the results of the analysis of variance (ANOVA) for the models of EY, GA, and DE. The quadratic polynomial models were well-fitted with the given data, and the projected models were obviously significant (low *p*-value 0.001 and substantial lack-of-fit) (Orellana-Palacios et al., 2022). On the other hand, EY, GA, and DE of TPP also had a very high determination coefficient for responses (98.72%, 98.69%, and 94.88%), thus showing that the suggested models can exactly and accurately describe the link between variables and responses (Bitaraf et al., 2012). The findings below indicate that higher-order models are not necessary since the proposed quadratic polynomial models are fully sufficient to predict the connection between independent variables and responses. All EY, GA, and DE of TPP using MAHE were carried out by multiple regressions to fit the second-order polynomial equation as following Eqs. (11−13).

(11) $$\mathrm{EY}\%=27.53+0.61\times {\;}_{1}+1.54\times {\;}_{2}+1.15\times {\;}_{3}–0.82\;X_{1}X_{2}–0.45\;X_{1}X_{3}+2.75\;X_{2}X_{3}–2.95\times {{}_{1}}^{2}–1.25\times {{}_{2}}^{2}+0.47\times {{}_{3}}^{2}$$

(12) $$\mathrm{GA}\%=62.25+12.04\times {\;}_{1}+6.21\times {\;}_{2}+4.97\times {\;}_{3}+2.65\;X_{1}X_{2}–1.73\;X_{1}X_{3}+1.43\;X_{2}X_{3}–2.65\times {{}_{1}}^{2}–7.25\times {{}_{2}}^{2}+0.42\times {{}_{3}}^{2}$$

(13) $$\mathrm{DE}\%=51.20+7.91\times {\;}_{1}–8.68\times {\;}_{2}–2.34\times {\;}_{3}–4.10\;X_{1}X_{2}–2.12\;X_{1}X_{3}+0.65\;X_{2}X_{3}–2.16\times {{}_{1}}^{2}+6.06\times {{}_{2}}^{2}–4.46\times {{}_{3}}^{2}$$

**Table 2:** Statistical information for yield, GA, and DE, regression results for the responsive surfaces, and quadratic model for all the independent factors.

Source	Yield %		GA %		DE %	
	Coefficient	p-value	Coefficient	p-value	Coefficient	p-value
Intercept	27.53	< 0.0001	62.25	< 0.0001	51.20	0.0031
X_1_-pH	0.6125	0.0116	12.04	< 0.0001	7.91	0.0009
X_2_-Temperature	1.54	0.0001	6.21	0.0002	−8.68	0.0005
X_3_-Time	1.15	0.0005	4.97	0.0005	−2.34	0.1197
X_1_ X_2_	−0.8250	0.0142	2.65	0.0438	−4.10	0.0655
X_1_ X_3_	−0.4500	0.1117	−1.73	0.1487	−2.12	0.2880
X_2_ X_3_	2.75	< 0.0001	1.43	0.2202	0.6500	0.7336
X_1_^2^	−2.95	<0.0001	−2.65	0.438	−2.16	0.2804
X_2_^2^	−1.25	0.0021	−7.25	0.0004	6.06	0.159
X_3_^2^	−0.4750	0.0968	0.4250	0.2202	−4.46	0.0499
Lack of fit		0.4342		22.53		73.16
C·V (%)	1.88		3.62		7.16	
R^2^	0.9872		0.9869		0.9488	
Adj R^2^	0.9681		0.9671		0.8721	
Adeq precision	30.99		23.4221		13.6476	

#### 3.1.1 The effect of extraction conditions on EY

**Fig. 1:**
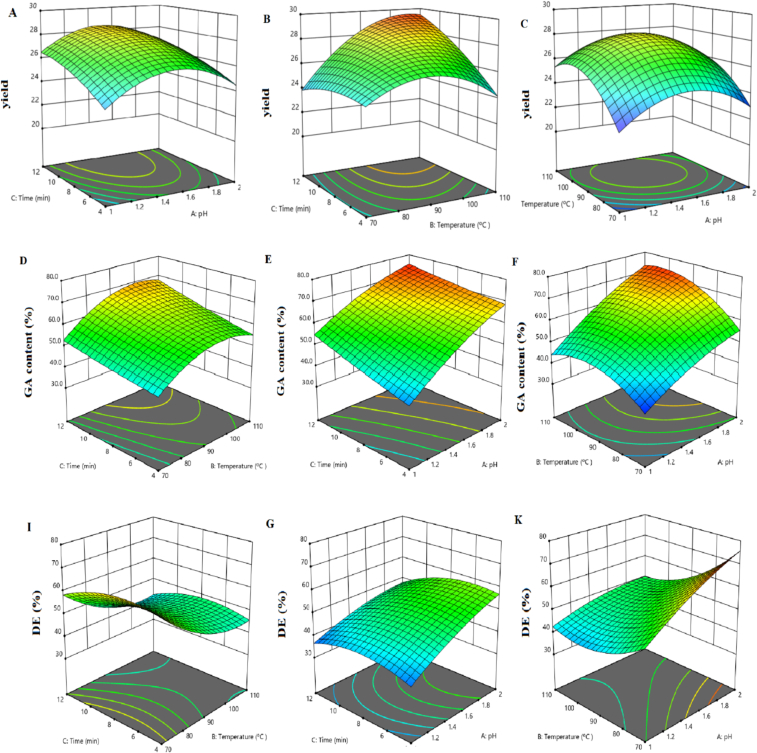
Impact of irradiation time, temperature, and pH on the EY (A, B, C), GAC (D, E, F), and DE (I, G, K) of TPP.

The yield of TPP varied between 20% (run = 13) and 32% (run = 7), as shown in Table 1, and this variation was found to be related to the pH, irradiation time, and temperature (Fig. 1). In Table 2, the results of ANOVA analysis indicate that irradiation time and temperature play critical roles in determining the extraction efficiency (p-value <0.001). Notably, the results revealed a positive correlation between EY and irradiation time, wherein an increase in irradiation time from 4 to 12 min resulted in an enhanced EY (Fig. 1A, B). This observation is probably attributed to increasing the expose time of the TP against microwave irradiation and hence, enhancing EY with a steep slope (Kazemi et al., 2019). Moreover, the use of a high power, specifically 1500 W, during MAHE, in combination with high pressure conditions, was found to significantly increase penetration of the extraction solvent into the pectin source matrix. This is achieved as a result of efficient molecular interactions with the electromagnetic field, which facilitate rapid energy transfer to the extraction solvent matrix. As a result, the constituents of interest within the matrix undergo effective dissolution and subsequent extraction (Liu et al., 2010). Temperature is also an important factor, with the EY increasing at higher temperatures and pressures (> 5 bar), especially when the temperature exceeds the boiling point of water (100–110 °C) in runs 1, 3, 7, 8 (Duan et al., 2022). Under those conditions, an increase in pressure from 5 to 10 bars enhances plant cell wall disruption (Fig. 1B, C). Similarly, the pH was identified as a crucial factor affecting the extraction yield of pectin, thus meaning that an appropriate pH must be selected to achieve maximum TPP extraction. As depicted in Fig. 1A and C, it is evident that the yield increased with decreasing pH, except under extremely harsh process conditions (pH < 1.0). Extraction solvents possessing high acidity were able to effectively mix with insoluble pectin, thereby promoting the hydrolysis of pectin constituents and facilitating increased pectin extraction. However, it should be noted that pectin degradation occurs under excessively harsh conditions, thus leading to a reduction in yield (Oliveira et al., 2016).

#### 3.1.2 The effect of extraction conditions on GAC

The purity of pectin is commonly assessed using the galacturonic acid content (GAC), as defined by (Wahengbam et al., 2014). In accordance with the regulations set by the Food and Agriculture Organization (FAO), pectin should contain a minimum of 65% galacturonic acid (Willats et al., 2006). Based on Eq. (12) and the results obtained from the analysis of variance (ANOVA), it was determined that pH had the most significant impact on GAC. As illustrated in Fig. 1E, F, higher pH levels were found to promote the formation of pectin with a high GAC. Conversely, harsh process conditions with pH values below 1.0 were observed to accelerate the degradation of pectic polysaccharides, thus leading to a decrease in pectin purity (Oliveira et al., 2016). The influence of irradiation time and temperature on GAC was relatively limited compared to pH. However, previous reports, as well as Fig. 1D, E, suggest that increasing irradiation time is associated with an increase in GAC (Bagherian et al., 2011). These findings align with studies conducted on sugar beet (Ma et al., 2013) and fresh citrus pectin (Hosseini et al., 2016), further supporting the results obtained in this study.

#### 3.1.3 The effect of extraction conditions on DE

Throughout the experimental studies, the degree of esterification (DE) of TPP ranged from 32.4% (run 6: pH 1.5, time = 12 min, and temperature = 90 °C) to 74% (run 14: pH 2, time = 8 min, and temperature = 70 °C), as shown in Table 1. Notably, the main variable affecting DE was found to be the pH (*p*-value <0.001). A reduction in DE for the pectin extracted was observed upon lowering the pH (Fig. 1G, K). Additionally, the results indicate that prolonged irradiation times and elevated temperatures contribute to a decrease in DE. Under such harsh conditions (low pH, long irradiation time, and high temperature), it is likely that the esterification of galacturonic acid (GA) chains is mainly responsible for the observed reduction in DE (Pasandide et al., 2017). This result supports the findings from run 14 (74%), which showed that the pectin isolated had a high level of esterification. Depending on these values, if DE > 50%, the extracted pectin is classified as highly esterified pectin, which can form rapid-set gels (Ann et al., 2015). Additionally, the results obtained in this study align with previous findings on grapefruit peel pectin extracted using either traditional heating (69.03%) or ultrasound-assisted extraction (58.78%). Similarly, Chinese orange pectin (DM = 74.51%) exhibited comparable outcomes when the peel was subjected to vacuum-microwave drying (Dong et al., 2021). Conversely, in runs 3, 4, 6, 7, 8, and 9 of the present study, the DE values were found to be below 50%. Pectin samples with lower DE values can be effectively utilized in dairy products and low-calorie meals given their distinctive functional properties (Zhao et al., 2023).

### 3.2 Comparison of extracted pectin using MAHE and CE methods

To determine the effectiveness of MAHE, a comparative analysis with CE was performed, as shown in Table 3. Of these two extraction techniques, MAHE had a significantly higher extraction yield (30.4 ± 0.64%) than CE (23 ± 0.74%), and the irradiation time required for MAHE (12 min) was shorter than for CE (120 min). As the yield is higher than that obtained by CE, this shows that the use of MAHE has a beneficial influence on the extraction of bioactive chemicals. This could be due to the fact that the TP cell wall is damaged more by a combination of microwave radiation and pressure (5 bar) than during CE, which may improve pectin release and increase the EY. This finding is consistent with previous research comparing pectin extraction methods, including extraction from satsuma mandarin peel using high hydrostatic pressure (Duan et al., 2022), hot-compressed water extraction from *Flos Magnoliae* (L. Wang et al., 2022), and a combined surfactant- and microwave-assisted process (Su et al., 2019). Furthermore, compared to the CE method, the MAHE method resulted in pectin with significantly (*p* < 0.05) improved EY (30.4 ± 0.64%), GA content (72.3 ± 0.75%), and EQ. W (1950.39 ± 1.21 g/mol). In addition, MAHE resulted in a lower protein content and DE (*p* < 0.05) (3.4 ± 0.86%), (45.2 ± 2.75%) than CE (4.3 ± 0.75%), (72.4 ± 2.83%), respectively. There were no significant changes in GAC for MAHE (GAC = 72.3 ± 2.48%) and CE (GA = 74.2 ± 2.13%), thus indicating that different extraction conditions had a less noticeable effect on the GAC.

**Table 3:** Qualitative and quantitative properties of pectin extracted using the two techniques.

Sample	PY (%)	GA content (%)	DE value (%)	g/mol	MC %
MAHE	30.4 ± 1.64	72.3 ± 2.48	45.2 ± 2.74	1950.3 ± 27	11.47 ± 1.21
CE	23.5 ± 1.74	74.2 ± 2.13	72.4 ± 2.83	625.6 ± 26	7.75 ± 1.38

In summary, MAHE with the acid treatment of TP may be of interest in the future for biorefinery feedstock, and this revolutionary technique can undoubtedly be used to extract a variety of bioactive chemicals from various natural sources quickly and efficiently (Matharu et al., 2016).

### 3.3 Equivalent weight (Eq.W) and methoxyl content (MC)

The equivalent weights (Eq.W) of pectin extracted using MAHE and CE were 1950.39 ± 1.21 and 626.63 ± 1.38 g/mol, respectively (Table 3). A higher Eq.W was observed for the pectin extracted using MAHE than for that extracted using CE. The ability of pectin to gel is determined by its molecular size and DE. Pectin Eq.W is an essential requirement for gelling ability, with a higher Eq.W indicating that an extracted pectin can create gel more easily (Israel et al., 2019). Table 3 shows that the MC of TPP varied depending on the extraction method (7.75 ± 1.38% for CE and 11.41 ± 1.21% for MAHE). In addition, the MC indicates the dispersibility of pectin in water and pectin gel formation (Ann et al., 2015). A high MC (usually 8–11%) can produce gels with a high sugar concentration (>65%), while a low MC (<7%) can result in the formation of gels with a low sugar level (Rouse et al., 1964).

### 3.4 Activity and Total phenolic content

#### 3.4.1 Total phenolic content (TPC)

**Fig. 2:**
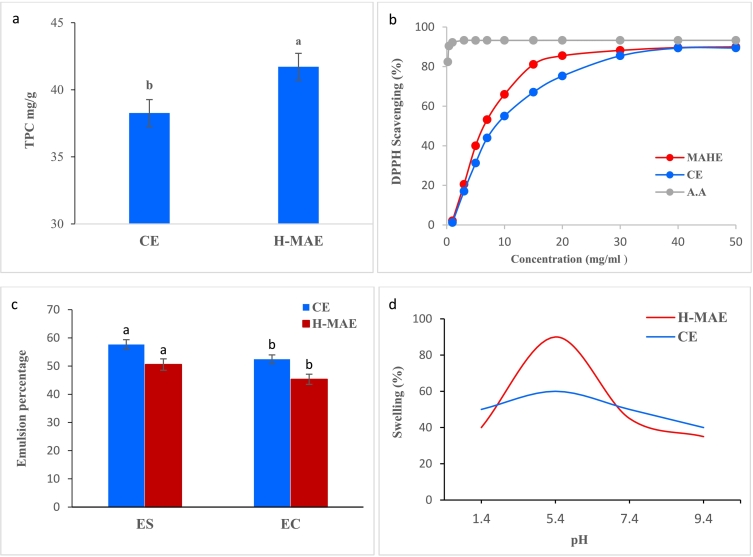
Antioxidant activity (a), total phenolic content (b), emulsifying properties (c), and swelling capacity (d) of TPPs in various pH buffer solutions. *Different letters in each column indicate significant differences between the different samples (*p* < 0.05).

Phenolic compounds are able to scavenge hydrogen atoms or electrons and bind metal ions to stabilize free radicals, thus meaning that they can act as antioxidants (Haghani et al., 2021). These properties mean that they can protect dietary products, especially oils and fatty acids against oxidation (Hadidi et al., 2022; Yancheshmeh et al., 2022). In this study, the Folin–Ciocalteu technique was used to calculate the TPC for the two methods: MAHE and CE. This technique is one of the most important methods for determining antioxidant properties. As shown in Fig. 2a, the TPC for TPP extracted under optimal extraction conditions using MAHE (pH 1.7, irradiation time = 12 min, and temperature = 108 °C) was 41.2 ± 1.67 mg gallic acid equivalents per gram of TPP (GAE/g) and 38.4 ± 1.23 mg GAE/g for CE. Although there are very few reports concerning the TPC of tangerine pectin, the results obtained for MAHE and CE herein are significant (p < 0.05) and better than for mandarin pectin (Sharma et al., 2015) and similar to those for pistachio green hull pectin (Kazemi et al., 2019).

#### 3.4.2 Antioxidant activity

DPPH radical scavenging, one of the most useful methods for assessing antioxidant activity, was used to determine the antiradical activity for the TPP obtained under optimal MAHE conditions (pH 1.7, irradiation time = 12 min, temperature = 108 °C) and compared to that for CE. Fig. 2b displays the results obtained, which demonstrate that increasing TPP concentration (sample concentration 50 mg/mL) improved the antiradical activity, which was approximately equal to the antiradical activity of ascorbic acid used as a reference. In addition, the antiradical activity for TPP was higher than that for pectin obtained from *Opuntia ficus indica* (OFI) cladodes at a concentration of 10 mg/mL (Bayar et al., 2017) but less than for pistachio green hull pectin (Kazemi et al., 2019). The effective antiradical action of TPP probably arises due to its total phenolic and GA content in addition to its low molecular weight (Bayar et al., 2017).

### 3.5 Techno-functional properties

#### 3.5.1 Emulsifying properties

**Fig. 3:**
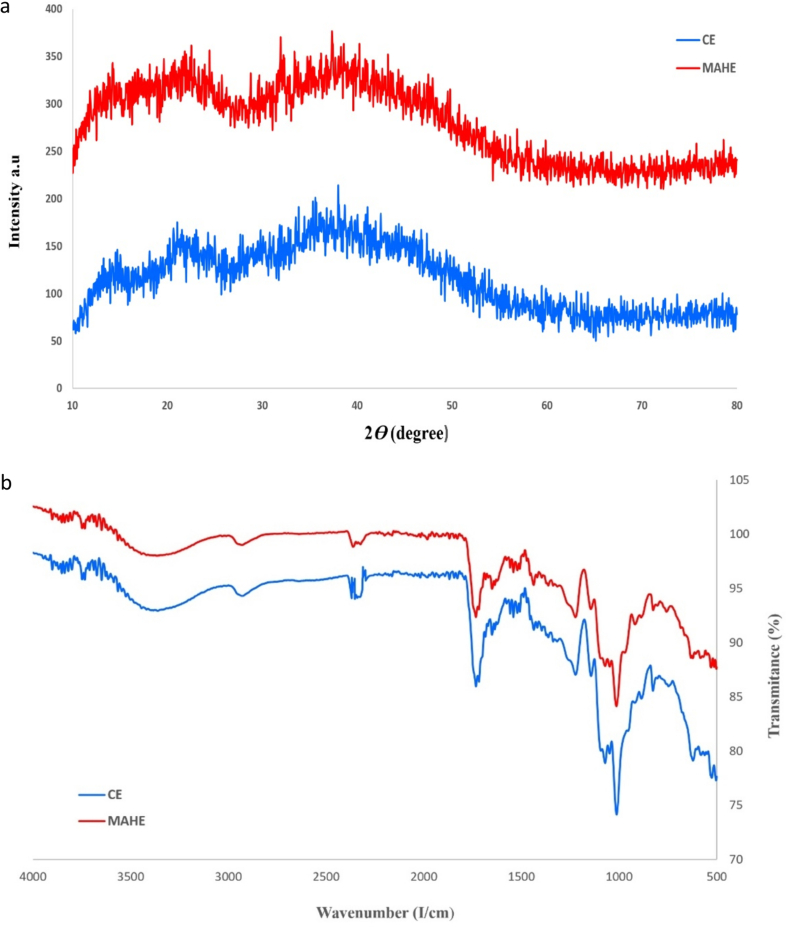
X-ray diffraction (XRD) (a) and Fourier transform infrared (FT-IR) spectra (b) for TPPs obtained under optimal MAHE and CE conditions.

In this experiment, the emulsifying activity (EA) and emulsifying stability (ES) of pectin extracted under optimum MAHE (pH 1.7, irradiation time = 12 min, and temperature = 108 °C) and CE conditions were examined using an emulsion created with pectin solutions (0.5% *w*/w). Fig. 2c shows that the emulsions formed with pectin have EA values of 57.65 ± 1.64% and 50.56 ± 1.77% for CE and MAHE conditions, respectively**,** the results obtained for MAHE and CE herein are no significant (*p* < 0.07), higher than those for citrus peel pectin extracted under optimal conditions by Hosseini et al. (Hosseini et al., 2016). The ES is clearly dependent on the pectin-extraction process, as shown in Fig. 3C. In fact, after 30 min of incubation at 80 °C the TPP extracted under CE conditions maintained >52.41 ± 1.54% of the emulsion, whereas the TPP extracted under MAHE conditions maintained around 39.32 ± 1.23%, the results are significant (*p* < 0.001). This is important durability for emulsions incubated at 80 °C might be attributed to an increase in solution viscosities generated by the creation of a layer of pectin around each oil droplet, which delays the coalescence process (Prajapati et al., 2013). The ES values in this investigation were substantially lower than the value for pectin extracted from OFI cladodes using the water-acid procedure at a concentration of 2% (90.45%) (Bayar et al., 2016). This reduction in ES for the same pectin might be related to a difference in extraction procedures. Indeed, the extraction technique may impact the average molecular weight and GA concentration, both of which affect emulsion stability (Funami et al., 2011).

#### 3.5.2 Swelling capacity (SC)

The SC of TP pectin extracted using the MAHE and CE techniques was studied in 20 mL buffer solutions at pH 1.5, 5.5, 7.5, and 9.5. The degree of swelling increased from pH 1.4 to 5.5, but then decreased from pH 5.4 to 9.4 (Fig. 3d). The highest swelling in solution (90%) is mainly due to complete ionization of the carbonyl group in pectin. The decrease in swelling after pH 5.5 is due to partial dissolution of the pectin species (M. Kumar et al., 2010). Pectin exhibits a strong pH sensitivity and superabsorbent properties. As such, this polymer could be used in a variety of biological applications such as thickening and hydration freshness preservation (Yi Hui Toy et al., 2022).

### 3.6 XRD analysis

The XRD pattern of TPP extracted under optimal-MAHE and CE conditions was recorded to determine the pectin structure (amorphous or crystalline). As can be seen in Fig. 3a, the XRD spectra show several sharp and intense peaks at 17, 18, 20, 24, 28, 31, and 37° for MAHE and 17, 18, 19, 21, 24, 29, 31, and 36° (2*θ*) for CE, which are due to its crystallinity. However, the spectra also exhibit an amorphous structure. Thus, it was found that the TPPs from MAHE and CE have both crystalline and amorphous components. This result is in agreement with the XRD results for pectin extracted by (Kazemi et al., 2019; Momeni et al., 2018).

### 3.7 FT-IR spectroscopy

The IR spectra of TPP extracted under MAHE and CE conditions are illustrated in Fig. 3b. The hydroxy groups and intramolecular hydrogen bonds are responsible for the bands at 3392 and 3393 cm^−1^ for MAHE and CE, respectively. The bands at 2860–2980 cm^−1^ are due to C—H stretching vibrations, including CH, CH_2_, and CH_3_ bending movements (W. Wang et al., 2016). The peaks at 1648, 1650 and 1731, 1732 cm^−1^ correspond to free (-COO) and esterified carboxylic groups (-COOR), respectively (Grassino et al., 2016). As shown in Fig. 3b, the peak for the -COOR groups is more intense for CE than MAHE, which is in agreement with the results obtained previously. The peaks between 1230 and 950 cm^−1^ are attributed to carbohydrates, whereas the bands around 1118, 1142 and 1222, 1219 cm^−1^ for MAHE and CE, respectively, were assigned to glycoside C-O-C vibrations (Hosseini et al., 2016). The strong peaks at 1013, 1049 and 1012, 1071 cm^−1^, are assigned to the high HG content (especially C—O, C-CH, C—C, and OCH vibrations) (Kpodo et al., 2017).

### 3.8 Thermogravimetric analysis (TGA)

**Fig. 4:**
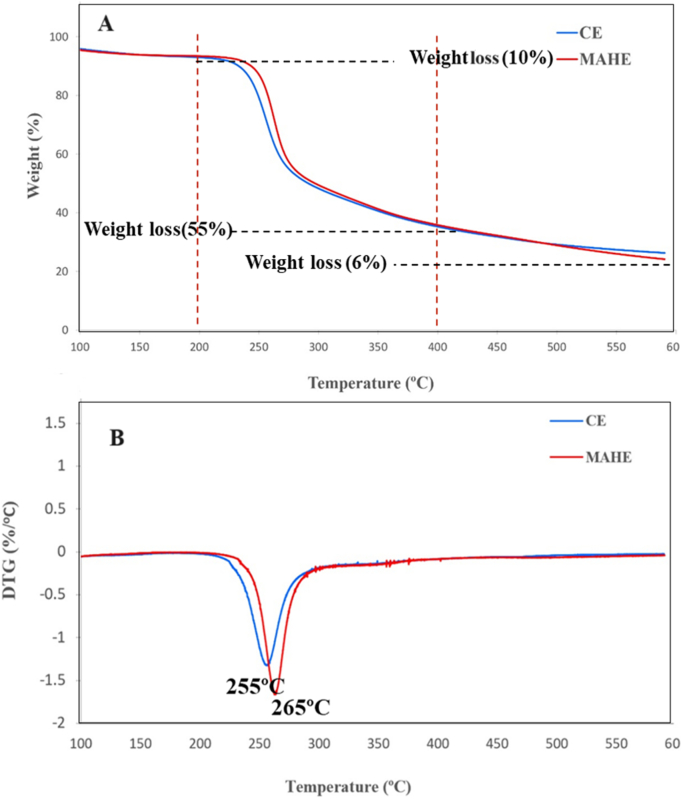
TGA (A) and DTG (B) thermograms for TPPs obtained under optimal MAHE and CE conditions.

TGA was used to further understand the thermal characteristics of MAHE TPP and CE TPP. Fig. 4A, B shows three different TGA ranges, namely 50–200 °C, 200–400 °C, and 400–600 °C, as also documented in previous studies (M. Kumar et al., 2010). The first range (50–200 °C) shows a small mass loss (approximately 10%) due to the evaporation of water from the pectin. Pyrolytic polysaccharide degradation causes a large weight loss (about 55%) in the second range (200–400 °C). At this point, substantial thermal degradation of the galacturonic acid chain of pectin occurs, as does decarboxylation of the acid side chains and carbon in the ring. These processes lead to the formation of gaseous products and solid char (Grassino et al., 2016). Finally, the third range, from 400 to 600 °C, reflects a progressive weight loss (about 6%) due to thermal decomposition of the char.

### 3.9 ^1^H NMR spectroscopy

**Fig. 5:**
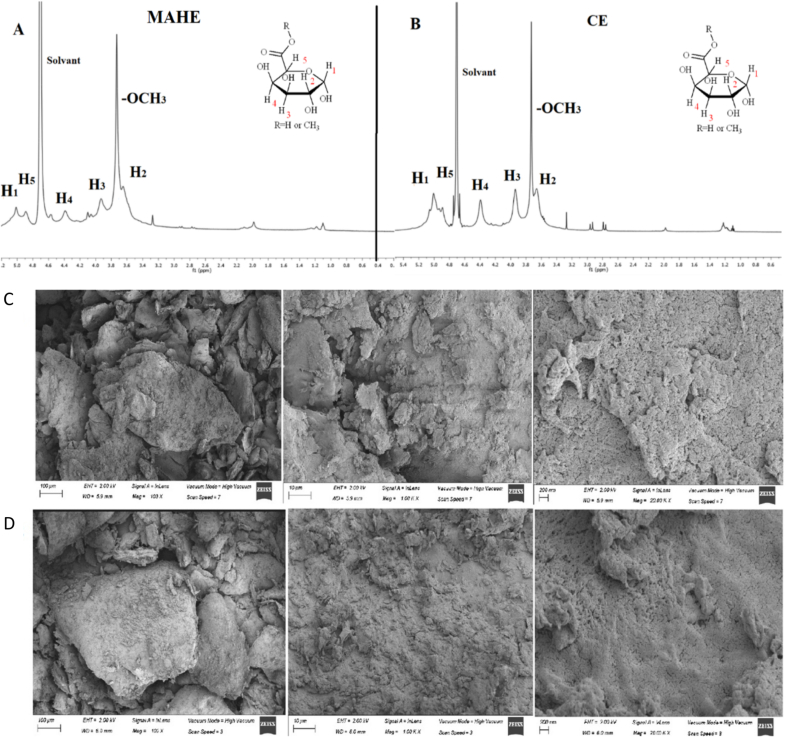
^1^H NMR spectra for optimal-MAHE (A) and CE (B) TPPs and Scanning electron micrographs for TPPs obtained by CE (C) and optimal-MAHE (D).

The ^1^H NMR spectra of TPP extracted using the MAHE (pH 1.7, time = 12 min, and temperature = 109 °C) and CE methods are shown in Fig. 5A,B, with most of the proton signals appearing at around 3.2 and 5.1 ppm. The methoxyl group protons of the esterified galacturonic acid units exhibit a large and sharp signal at 3.7 ppm. The signals at 4.9 (H-5) and 5.0 (H-1) ppm are due to non-esterified GA units (Sharma et al., 2015). Galacturonate and methyl galacturonate residues exhibit proton signals for H-1, H-2, H-3, and H-4 at 5.0, 3.6, 3.9, and 4.4 ppm, respectively. The methyl group linkages of rhamnose are probably responsible for the signals observed at roughly 1 ppm (Kpodo et al., 2017), and the signals at 2 ppm are most probably due to the acetyl groups (CH_3_). The above results confirm the presence of the pectin structure in the extracted sample and agreed with previous studies (Grassino et al., 2016; Kpodo et al., 2017; Sharma et al., 2015).

### 3.10 Morphology surface

A scanning electron microscope (SEM) was used to assess the impact of the extraction method on the surface morphology of pectin samples produced using the optimized MAHE and CE methods. Previous studies have found that the surface of MAHE pectin is normally very rough and somewhat ruptured. The quick increase in temperature and the high internal pressure associated with the MAHE procedure appear to have an effect on the morphology (Dranca et al., 2020). Fig. 5 C, D shows SEM images of pectin acquired in this study. The microstructures of the samples recovered after CE (B) and MAHE (C) vary significantly. Thus, whereas TPP extracted by CE shows a slight tendency to curl, the TPP extracted by MAHE seems to be ruptured. Based on the findings of this study, the MAHE technique appears to change the surface morphology of pectin. This can probably be explained by the sudden increase in temperature and internal pressure during the extraction process, as suggested previously (Liew et al., 2016).

## 4 Conclusion

In this study, we employed microwave-assisted hydrothermal extraction (MAHE) to extract pectin from tangerine peels, which are widely recognized as a valuable source of bioactive compounds. The extraction process was optimized using a Box–Behnken design for statistical analysis. Under the optimal conditions identified (pH 1.7, *t* = 12 min, and temperature = 109 °C), we achieved a maximum yield of tangerine peel pectin (TPP) of 30 ± 1.64%. The degree of esterification (DE) was below 50%, specifically 45.2 ± 2.74%, thus indicating the suitability of the pectin extracted for applications in dairy and low-calorie meals. The galacturonic acid (GA) content was notably high, at approximately 72.3 ± 2.83%. In terms of physicochemical and structural properties, the MAHE-extracted pectin exhibited a total phenolic content of 41.2 ± 1.67 mg GAE/g pectin. The equivalent weight of the pectin was measured to be 1950.31 ± 2.78 g/mol, while the methoxyl content was 11.47 ± 1.21%. Importantly, the TPP demonstrated emulsifying activity with a value of 50.56 ± 3.77%, thus indicating its potential as a suitable emulsifier. The presence of esterified pectin in the samples obtained was confirmed by ^1^H NMR and FTIR spectroscopic analysis. Additionally, SEM and XRD measurements revealed that the pectin extracted via MAHE under optimal conditions exhibited a hard texture and a crystalline structure. A comparison of this pectin with that obtained by conventional extraction (CE), under the same optimal conditions, showed that MAHE provided a 7.0% improvement in extraction yield (EY) while maintaining superior physicochemical and functional properties. As a result, MAHE may be a promising alternative technique that offers multiple advantages, such as shorter extraction times, higher yields, improved pectin quality, enhanced swelling and holding capacity, and reduced energy requirements. Finally, MAHE has considerable potential as a feasible replacement for conventional extraction techniques.

## CRediT authorship contribution statement

**Imed E. Benmebarek:** Writing – original draft, Software, Methodology, Investigation, Formal analysis, Data curation. **Diego J. Gonzalez-Serrano:** Writing – original draft, Software, Methodology, Investigation, Formal analysis, Data curation. **Fatemeh Aghababaei:** Data curation, Formal analysis, Investigation, Writing – original draft, Writing – review & editing. **Dimitrios Ziogkas:** Writing – original draft, Methodology, Investigation, Formal analysis, Data curation. **Rosario Garcia-Cruz:** Investigation, Formal analysis, Data curation. **Abbas Boukhari:** Supervision. **Andres Moreno:** Visualization, Validation, Supervision, Resources, Conceptualization. **Milad Hadidi:** Writing – review & editing, Writing – original draft, Visualization, Validation, Supervision, Resources, Conceptualization.

## Declaration of competing interest

The authors declare that they have no known competing financial interests or personal relationships that could have appeared to influence the work reported in this paper.

## Data Availability

Data will be made available on request.
